# Do motor control genes contribute to interindividual variability in decreased movement in patients with pain?

**DOI:** 10.1186/1744-8069-3-20

**Published:** 2007-07-26

**Authors:** Bikash K Mishra, Tianxia Wu, Inna Belfer, Colin A Hodgkinson, Leonardo G Cohen, Carly Kiselycznyk, Albert Kingman, Robert B Keller, Qiaoping Yuan, David Goldman, Steven J Atlas, Mitchell B Max

**Affiliations:** 1 Clinical Pain Research Section, Laboratory of Sensory Biology, National Institute of Dental and Craniofacial Research, National Institutes of Health, DHHS, Bethesda, MD, USA; 2 Laboratory of Neurogenetics, National Institute on Alcohol Abuse and Alcoholism, National Institutes of Health, DHHS, Rockville, MD, USA; 3 Statistics Core, Division of Population and Health Promotion Sciences, National Institute of Dental and Craniofacial Research National Institutes of Health, DHHS, Bethesda, MD, USA; 4 Human Cortical Physiology Section, National Institute of Neurological Diseases and Stroke, National Institutes of Health, DHHS, Rockville, MD, USA; 5 Maine Spine and Rehabilitation, Portland, ME, USA; 6 General Medicine Division and the Clinical Epidemiology Unit, Medical Services, Massachusetts General Hospital, Harvard Medical School, Boston, MA, USA

## Abstract

**Background:**

Because excessive reduction in activities after back injury may impair recovery, it is important to understand and address the factors contributing to the variability in motor responses to pain. The current dominant theory is the "fear-avoidance model", in which the some patients' heightened fears of further injury cause them to avoid movement. We propose that in addition to psychological factors, neurochemical variants in the circuits controlling movement and their modification by pain may contribute to this variability. A systematic search of the motor research literature and genetic databases yielded a prioritized list of polymorphic motor control candidate genes. We demonstrate an analytic method that we applied to 14 of these genes in 290 patients with acute sciatica, whose reduction in movement was estimated by items from the Roland-Morris Disability Questionnaire.

**Results:**

We genotyped a total of 121 single nucleotide polymorphisms (SNPs) in 14 of these genes, which code for the dopamine D2 receptor, GTP cyclohydrolase I, glycine receptor α1 subunit, GABA-A receptor α2 subunit, GABA-A receptor β1 subunit, α-adrenergic 1C, 2A, and 2C receptors, serotonin 1A and 2A receptors, cannabinoid CB-1 receptor, M1 muscarinic receptor, and the tyrosine hydroxylase, and tachykinin precursor-1 molecules. No SNP showed a significant association with the movement score after a Bonferroni correction for the 14 genes tested. Haplotype analysis of one of the blocks in the GABA-A receptor β1 subunit showed that a haplotype of 11% frequency was associated with less limitation of movement at a nominal significance level value (p = 0.0025) almost strong enough to correct for testing 22 haplotype blocks.

**Conclusion:**

If confirmed, the current results may suggest that a common haplotype in the GABA-A β1 subunit acts like an "endogenous muscle relaxant" in an individual with subacute sciatica. Similar methods might be applied a larger set of genes in animal models and human laboratory and clinical studies to understand the causes and prevention of pain-related reduction in movement.

## Background

Pain-evoked limitation of activities is one of the most costly morbidities of illness [1]. Several studies have replicated the finding that bed rest or a decrease in activity may impair recovery from an acute back pain episode [2-5]. Clinical practice guidelines recommend early activity in the management of acute low back pain [6,7].

If decreased activity in the presence of back pain retards recovery, it may be important to understand why some patients decrease their activity more than others after a similar injury. Most of the relevant work to date has focused on psychological causes of reductions in motor activity in pain patients [8,9] paying relatively little attention to factors intrinsic to the motor system or direct connections with afferent pain systems. For example, Lethem et al [8] proposed the fear-avoidance model to explain a possible mechanism of inactivity and disability in patients with chronic musculoskeletal pain. According to this hypothesis, if an individual misinterprets back pain as a signal of reinjury or continued tissue damage, his fear of injury may cause him to systematically avoid movements that typically increase pain [10]. The resulting inactivity may lead to deconditioning, contractures, and disability.

Psychological variables almost certainly explain some of the variation in the motor response to injury, but we propose that one should also examine the contribution of inter-individual differences in the wiring and neurochemistry of the motor system itself. Lund et al. [11] have proposed a plausible link from pain inputs to motor circuits, the "pain-adaptation model." In this model, pain afferent activity decreases activity of the muscle groups that move a joint in the pain-provoking direction and increases the activity of muscle groups that antagonize such movement. These motor adjustments reduce movement velocity and limit excursions, thereby protecting against pain.

One way to examine potential differences in motor control processes is to test for polymorphisms in genes encoding molecules that regulate motor systems. We and others have already been studying the association between short lists of candidate genetic polymorphisms and the severity of acute and chronic pain [12-14] and pain-related mood change [15]. Arrays of 500,000 or more single nucleotide polymorphisms are commercially available, making possible whole genome association studies in large cohorts of pain patients. It occurred to us that in cohorts where one had a good measure of motor function, one could use the same whole genome scan data to search for genes that predisposed to a greater decrease in activity, given similar injury and pain level. Many current spine pain studies will include enough data to make this possible, because a decade ago, leading spine researchers [16] agreed that studies should routinely incorporate either the Roland-Morris [17] or Oswestry Disability questionnaires [18], which question the patient about many motor functions.

We propose that there may be genetic polymorphisms that directly affect the motor system to cause a maladaptive level of guarding and hypoactivity. In preparation for our studies to test this hypothesis, we have examined the literature on motor systems to suggest some candidate genes. We previously proposed a method for prioritizing genetic polymorphisms for clinical studies in which pain intensity is the primary endpoint [19]. We have adapted this into a method to prioritize polymorphisms that may contribute to reduction in movement in the presence of pain, and will describe this method and our resulting list of plausible candidate genes.

## Results

### High priority motor control candidate genes

The prioritization process generated the candidate gene list shown in Table 1, plus additional genes, not shown, that scored lower than those in Table 1. Our literature search showed that there was virtually no work specifically on the neurochemistry of pain-related motor changes in mammalian models. Therefore, the ratings for "relation to motor control" just reflect the degree of certainty that the molecule is present at key sites in motor control systems, from basal ganglia down to the neuromuscular junction.

**Table 1:** High-priority candidate polymorphisms that may affect motor control.

Gene*	**Molecule**	**SNP**	**Location**	**AA change**	**Ref**	**Frequency**		**Function**	**Relation to Motor Control**	**Total**
						**%**	**No**			
**DRD2/ANKK1**	Ankyrin repeat and kinase domain 1	C32806T	3' region/Exon 8	Glu713 Lys	[42]	46	3	3	3	9
**GCH1**	GTP cyclohydrolase 1	C94A	Exon 1	Thr 94 Lys	[14]	15	2	3	3	8
**GLRA1**	Glycine receptor 1α	G1192A	Exon 6	Arg271 Glu	[43]	12	2	3	3	8
CACNA1S	Calcium channel, voltage-dependent, L type, α-1S subunit	T2403C	Exon 18	no	[44]	25	3	2	3	8
GABR1A	GABA A receptor 1 α	T156C	Exon 4	Lys278 Met	[45]	28	2	3	3	8
RyR1	Ryanodine receptor 1 gene	G6178T	Exon 38	Gly2060 Val	[46]	12	2	3	3	8
**ADRA2C**	Adrenergic receptor 2C	12 NT Ins/Del	Coding region/3rd intracellular loop	no	[47]	35	3	2	2	7
**5HT2A**	Serotonin 2A receptor	T102C	Exon 3	His 452 Tyr	[48]	9	1	3	3	7
GABBR1	GABAB receptor gene 1	G1465A	Exon 11	Gly489 Ser	[49]	10	2	2	2	6
**ADRA2A**	α2A adrenergic receptor	C1291G	Promoter region	no	[50]	26	2	2	2	6
**5HT1A**	Serotonin 1A receptor	C1019G	Promoter region	no	[51]	29	2	2	2	6
**CNR1**	Cannabinoid receptor gene 1	G1359A	Exon1	no	[52]	31	3	0	2	5
**CHRM1**	Human M1 muscarinic receptor	C1221T	Exon 6	C407	[53]	16	2	1	2	5
Rab3A	Rat brain associated protein 3A	C428T	Exon3	Val72 Met	[54]	26	2	1	2	5
SCN4a	Sodium channel receptor gene	A669G	Exon 12	Arg672 Cys	[55]	NA	0	2	3	5

*Genes listed in bold type were genotyped in this study, along with 5 intermediate-priority polymorphisms

### Non-genetic factors contributing to movement reduction

**Figure 1:**
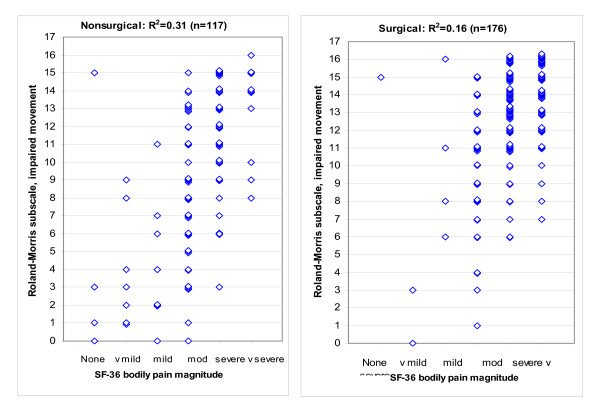
Impairment of movements involved in everyday activities on the day of study entry (Roland Morris Disability Questionnaire subscale, Table 2) vs. magnitude of "bodily pain" (SF-36) in the month prior to study entry. The patients are Maine Lumbar Spine Study sciatica cohort patients who subsequently chose nonsurgical (left) or surgical treatment (right). Some y axis values have been slightly altered to let the reader estimate the number of patients with overlapping data points. Not surprisingly, patients with more pain report limitation of more activities.

Table 2 shows the Roland-Morris Disability Questionnaire items that we included in the motor limitation subscale. Figure 1 shows that patients with higher SF-36 bodily pain magnitude ratings reported greater movement limitation. Pain rating contributed 31% of the variance in movement impairment in patients who chose nonoperative treatment, and 16% of the variance in those who chose surgery. Table 3 shows other non-genetic contributors to variation in movement impairment. For this and subsequent statistical analyses, we did not include pain after observing that a two-factor, *gene × pain *analysis of covariance for movement impairment yielded many more significant genetic associations than would have occurred by chance, even for lists of genes that were not on our motor candidate list. Our only explanation for this was that because the Roland-Morris movement questions include wording referring to both pain and movement, this might produce unanticipated biases of analyses that include both pain and movement. Therefore we simplified the analysis by recognizing that all patients presented to the study with leg and back pain – an entry criterion – and dropping pain magnitude from the analysis. This step corrected the number of SNP associations to the number expected by chance.

**Table 2:** Roland-Morris Disability Questionnaire.

**1**	**I stay at home most of the time because of my back problem or leg pain (sciatica).**
2	I change position frequently to try and get my back or leg comfortable.
3	**I walk more slowly than usual of my back problem or leg pain (sciatica).**
**4**	**Because of my back problem, I am not doing any of the jobs that I usually do around the house.**
**5**	**Because of back problem, I use a handrail to get to upstairs.**
**6**	**Because of my back problem, I have to hold onto something to get out of an easy chair.**
**7**	**I get dressed more slowly than usual because of my back problem or leg pain (sciatica).**
**8**	**I only stand for short periods of time because of my back problem or leg pain (sciatica).**
**9**	**Because of my back problem, I try not to bend or kneel down.**
**10**	**I find it difficult to get out of chair because of my back problem or leg pain (sciatica).**
11	My back or leg is painful almost all the time.
12	**I find it difficult to turn over the bed because of pain in my back or leg.**
**13**	**I have trouble putting on my socks (or stockings) because of the pain in my back or leg.**
14	**I only walk short distances because of my back or leg pain (sciatica).**
15	I sleep less well because of my back problem.
**16**	**I avoid heavy jobs around the house because of my back problem.**
**17**	Because of my back problem, I am more irritable and bad tempered with people than usual.
**18**	**Because of my back problem, I go upstairs more slowly than usual.**
**19**	**I stay in bed most of the time because of my back or leg pain (sciatica).**
20	Because of my back problem, my sexual activity is decreased.
21	I keep rubbing or holding areas of my body that hurt or are uncomfortable.
22	**Because of my back problem, I am doing less of the daily work around the house than I would usually do.**
23	I often express concern to other people over what might be happening to my health.

Note: In the Maine Lumbar Spine Study, patients were asked to answer yes or no to each question to describe their condition "today," at study entry after referral to a specialist for acute or subacute sciatica. The total score is the number of items answered "yes." We selected the items in bold type as a "movement impairment" subscale.

**Table 3:** Variables that contribute to baseline Roland-Morris motor limitation subscale score.

*Quantitative variable*	N	Mean	STD	Correlation (r) with baseline Roland-Morris		R^2^	p-value
	
SF-36 Vitality	309	40.00	21.74	-0.5384		0.2898	<0.0001
SF-36 Emotional Role	306	51.74	42.84	-0.3410		0.1163	<0.0001
Age	311	42.03	10.90	-0.0368		0.0014	0.5175
				Baseline Roland-Morris			
*Categorical variable*	Category		n	Mean	STD		
	
Med vs. surgical treatment	Surgical		183	12.38	3.12	0.1605	<0.0001
	Medical		128	9.02	4.60		
Workman's compensation	Yes		114	11.67	3.77	0.0154	0.0288
	No		197	10.60	4.29		
Marital status	Married/living together		244	11.00	4.18	0.0244	0.0223
	Never Married		29	9.38	4.35		
	divorced/separated/widow		38	12.18	3.30		
Neurological exam deficits	0		76	9.74	4.51	0.0431	0.0037
*each class*	1		106	10.98	4.36		
*(motor, sensory, reflex)*	2		97	11.64	3.50		
*counts for1*	3		31	12.45	3.21		
Sex	Male		189	11.01	4.17	0.0000	0.9505
	Female		122	10.98	4.09		

R-square from the model including all above eight variables (n = 302 patients, model df = 9)						**0.4420**	

Note: In the Maine Lumbar Spine Study, patients were asked to answer yes or no to each question to describe their condition "today," at study entry after referral to a specialist for acute or subacute sciatica. The total score is the number of items answered "yes." We selected the items in bold type as a "movement impairment" subscale.

Table 3 shows that greater reduction in movement was associated with lower scores for the SF-36 Vitality subscale (R^2 ^= 29%); the choice of surgical treatment (R^2 ^= 16%); the patient receiving workers' compensation (R^2 ^= 1.5%), the presence of neurological deficits on exam (R^2 ^= 4%), and lower scores, indicating greater impairment, on the SF-36 Emotional Role subscale (R^2 ^= 12%). A combination of all of the variables in the model explained 44% of the variance in the movement reduction subscale.

### Analysis of 14 movement candidate genes and movement reduction

Resources permitted us to genotype a set of SNPs from 14 of these genes: 9 of the top priority 15 genes in Table 1, and 5 lower priority genes that were included in the Laboratory of Neurogenetics' array of 130 polymorphic genes of interest to a consensus of neuropsychiatric and addictions researchers. No SNPs in the fourteen genes we tested – the dopamine D2 receptor, GTP cyclohydrolase I, glycine receptor α1 subunit, GABA-A receptor α2 subunit, GABA-A receptor β1 subunit, α-adrenergic 1C, 2A, and 2C receptors, serotonin 1A and 2A receptors, cannabinoid CB-1 receptor, M1 muscarinic receptor, and the tyrosine hydroxylase, and tachykinin precursor-1 molecules – showed a significant association with the movement score after a Bonferroni correction for the multiple candidate genes tested (Additional file 1). The two SNPs in the α-2C adrenergic receptor that were nominally significant in the additive model (without correction for multiple tests, Table 4) made small contributions to the variance in movement scores – approximately 0.6% and 0.9% (partial R^2^). Neither of these SNPs is known to cause a functional change in molecular function. Haplotype analysis of this gene using all four ADRA2C SNPs genotyped in this study showed a nominally significant association (p = 0.02) with movement scores which loses significance when one corrects for the 22 haplotype blocks examined.

**Table 4:** Association of four ADRA2C markers with movement scores in sciatica patients

**dbSNP ID**	**Allele1**	**Allele2**	**Genotype**	**Count**	**Movement Score**	*p *Value*
rs13118771	T	C	1/1	253	11.22	**0.0267**
			1/2	53	10.16	
			2/2	4	9.87	

rs6846820	**A**	G	1/1	6	9.19	**0.0182**
			1/2	51	10.26	
			2/2	253	11.23	

rs7434444	C	**G**	1/1	16	11.63	0.4313
			1/2	139	11.13	
			2/2	155	10.88	

rs7678463	**C**	G	1/1	6	9.35	0.2490
			1/2	74	10.84	
			2/2	230	11.12	

Haplotype analysis	No. of haplotype 2121 (CAGC)					
			2	3	9.3	**0.0216**
			1	52	10.2	
			0	255	11.2	

*p *values shown are for additive model, which assumes that each copy of the uncommon allele alters the movement score by a similar amount, and uncorrected for examining 121 SNPs in 22 haplotype blocks in 14 genes.

Haplotype analysis of one of the blocks in the GABA-A receptor β1 subunit showed that a haplotype of 11% frequency was associated with less limitation of movement at a significance level (p = 0.0025; Table 5) almost strong enough to correct for testing 22 haplotype blocks.

**Table 5:** Association of six GABRB1 markers with movement scores in sciatica patients

**dbSNP ID**	**Allele1**	**Allele2**	**Genotype**	**Count**	**Movement Score**	*p *Value*
rs4694846	A	**G**	1/1	125	11.35	0.1862
			1/2	136	10.85	
			2/2	49	10.81	

rs17461905	**A**	G	1/1	222	10.83	0.1219
			1/2	84	11.46	
			2/2	4	11.73	

rs13107066	**A**	C	1/1	103	11.17	0.8874
			1/2	136	10.84	
			2/2	71	11.09	

rs6813436	A	**G**	1/1	4	10.53	0.6383
			1/2	68	11.29	
			2/2	238	10.96	

rs7439087	**A**	G	1/1	148	10.73	0.2294
			1/2	131	11.29	
			2/2	31	11.19	

rs 6290	T	**C**	1/2	42	10.93	0.8447
			2/2	268	11.04	

Haplotype analysis	No. of haplotype 211212 (GAAGAC)					
			2	8	8.5	**0.0025**
			1	52	10.3	
			0	248	11.3	

*p *values shown are for additive model, which assumes that each copy of the uncommon allele alters the movement score by a similar amount, and uncorrected for examining 121 SNPs in 22 haplotype blocks in 14 genes.

Power analysis indicated that our study had sufficient power (85%) to detect effect of a single SNP that contributed 5% (partial R^2 ^= 0.05) of the movement variance after correcting for Bonferonni error using α = 0.00357 (=0.05 divided by the number of genes tested, 14).

## Discussion

Our literature review showed that the genes for many molecules associated with motor control circuits have common polymorphisms. Such polymorphisms might conceivably contribute to excessive guarding shown by some patients with pain.

The present study of 14 of these candidate genes revealed an association of a GABA-A β1 subunit haplotype with the amount of motor limitation (Table 5). Two copies of the haplotype reduced movement limitation scores almost 30% compared with no copies. The nominal p value of 0.0025 is small enough to be interesting even if one corrects for the 22 haplotype blocks examined, yielding an overall p value = 0.055. If confirmed by subsequent studies, we would hypothesize that this variant of the gene serves as an "endogenous muscle relaxant", with greater GABAergic motor inhibitory activity that reduces spasm and permits more activities.

We cannot speculate about the specific biochemical mechanism by which this haplotype might affect motor function. The finding that the haplotype analysis was much more sensitive than single SNP analyses suggests that a movement-facilitating SNP that we did not directly test may be present on the haplotype we identified. Compared to other GABA receptors, the GABA-A receptor β1 subunit has been little studied [20], and there is no commonly used specific antagonist, agonist, or knockout mouse. The published literature states that benzodiazepine muscle relaxant effects are primarily mediated through GABA-A α2, α3, and α5 receptor subunits [21]. However, interest in the β1 subunit is growing because of possible associations with autism [22] and bipolar disorder [23], so the gene may soon be better understood.

The nominally significant associations of two ADRA2C SNPs and a haplotype with motor limitation scores are not persuasive because of the modest size of the effect and the many genes tested. However, this gene is known to have common polymorphisms with strong effects on cardiovascular regulation and mood, such as a deletion affecting about 40% of chromosomes in African Americans [24,25], not tested for in the current study of a Caucasian population. Alpha-2 adrenergic receptors mediate inhibition of motor tone at the spinal level [26], so this gene should be revisited in future studies.

Because of several limitations in our study methods, we consider these findings tentative. The first limitation is that the Roland-Morris scale items that we used are retrospective and subjective assessments of the patient's ability to carry out various movements of daily life. Moreover, we chose a subset of these items to represent changes in movement without any formal validation studies. Objective measures of motor function would have been more convincing, but these were not available for this cohort.

Secondly, we tested only 14 genes, chosen by an anatomical analysis the emphasized the final two or three neurons in the motor control pathway. Molecules influencing more rostral levels of control might also be candidates. For example, Kleim et al. [27] have reported a BDNF polymorphism to be associated with modified plasticity in the human motor cortex. A more thorough search could be done using a whole genome association array, but this would require a sample size in the thousands [23] to provide adequate power.

Thirdly, we would have been interested in searching for an interaction between gene effect and pain level on movement, similar to the gene, pain, and depression analysis we have done on the same cohort [15]. For example, some polymorphic alleles might produce a major reduction in movement only in the presence of severe pain. However, this analysis was not appropriate in this case because the scale items mention both pain and various movements.

Finally, long-term decrease in movement is more directly relevant to the economic burdens of pain than the acute effects that we examined above. The analytical approach demonstrated above might also be used to search for effects of genes on a chronic decrease in movement. We chose to examine acute pain and movement data as an initial demonstration of this method for several reasons: (1) Almost all of our 290 patients who gave a DNA sample had pain at the baseline measurement, while a minority had persistent pain at one year or later time points. Sample size is crucial in screening multiple candidate genes [19]. (2) A shorter time window in which patients are studied under similar methods and treated by the same clinicians may be more sensitive to genetic effects than a longer period during which many types of environmental variability overshadow gene effects. (3) The processes involved in chronic pain and movement limitation are probably more complex than acute pain, including deconditioning, muscle atrophy and contractures, chronic psychiatric morbidity, and occupational factors.

Although physicians commonly prescribe "muscle relaxants" such as cyclobenzaprine, carisoprodol, and methocarbamol for acute pain, the database supporting their effects on muscle and clinical usefulness is limited [28]. Development of new treatments will require expansion of animal research on the neurochemical mechanisms of pain-related guarding. We propose that, as has been demonstrated in genetic studies of pain [14], genomic screens in humans may be useful in to prioritizing targets identified in animal studies.

## Conclusion

We have illustrated a method to use routinely measured variables in musculoskeletal pain studies to screen for molecules that may be associated with the excessive pain-related decrease in movement that increases risk of chronic disability. After correction for multiple comparisons, we found a modestly significant association between a haplotype in the GABA-β1 receptor subunit with motor limitation scores. Replication of this finding and the use of such methods with larger samples or direct measures of movement [29,30] might open up a new facet of the relationship between pain and movement.

## Methods

### Prioritization of "motor control" polymorphisms

Although muscle stiffness and reduction of movement in pain patients is widely discussed in the literature [31,32], there is no evidence regarding its molecular basis. Hence we reviewed the motor control research from animal and human studies to compile a list of molecules which appear to be involved in normal motor control and in diseases where muscle tone is altered and movement restricted and muscle stiffness.

We searched the literature for reports of the transmitters, receptors, and signaling molecules mediating control of limb and trunk movements, tone, and posture. We searched PubMed for review articles on motor control mechanisms and for citations of neurotransmitters, receptors, or signaling molecules relevant to motor cortex, brainstem, basal ganglia, cerebellum, locus coeruleus, corticospinal tract, ventral horn, interneuron, Renshaw cells, and neuromuscular junction. We prioritized polymorphisms in the genes for these molecules similar to the criteria proposed by Belfer et al. [19]. Three scores of 0–3 were assigned to each polymorphism according to (a) the strength of evidence supporting involvement of the gene in motor control; (b) population frequency of the polymorphism; and (c) strength of evidence that the polymorphism is associated with an altered clinical or molecular phenotype.

#### A). Strength of evidence supporting the involvement of gene in motor control and muscle tone regulation

We assigned one point if a single paper reported anatomical, physiological, or pharmacological evidence plausibly associating the molecule with motor control, two points for multiple reports, and three points for multiple reports that show alterations of that molecule alter muscle tone in animals or in patients.

#### B). Population frequency of polymorphism

Given equal effects on function, more common polymorphisms more efficient to study because there are sufficient numbers of patients with one or two copies of the uncommon allele. We assigned zero points if the population frequency of the variant is less than 3%, one point for 4–9%, two points for 10–29% and three points for 30–50%.

#### C). Functional effects of polymorphism

We made a PubMed and Panther database search and assigned one point if the variant changed an amino acid, two points for one report that the variant changes the amount of mRNA or protein expression or function or is associated with a clinical phenotype, and three points for independent replication of any of these types of evidence.

### Patients

Participants were members of the sciatica group in the Maine Lumbar Spine Study (MLSS [33]). The MLSS was a prospective cohort study conducted by approximately half of Maine's orthopedists and neurosurgeons who actively treated spine disease. Patients were enrolled between 1990 and 1992, and surgical discectomy or non-surgical treatment was chosen based upon clinician judgment and patient preference. Patients completed questionnaires at a baseline assessment, and then at 3, 6, and 12 month follow-up, and then annually through year 10. After completion of the 10 year study, the NIDCR and MLSS investigators developed a collaboration to add a genetic component to the study, under a protocol approved by the NIDCR Institutional Review Board. Patients were invited to contribute a DNA sample. Two hundred ninety of the original 507 enrollees provided DNA and had usable baseline data.

#### Pain measure

The primary measure of pain for this study was the Bodily Pain intensity item on the Short-Form-36 (SF-36) quality of life instrument [34] at baseline. Patients responded to the question "How much bodily pain have you had during the past 4 weeks?" by choosing from "very severe," "severe," "moderate," "mild," "very mild," and "none."

### Selection of "movement impairment subscale" from Roland-Morris

Table 2 lists the 23 items in The Roland-Morris Disability Questionnaire. We selected the 16 items that refer to a pain-related reduction in movement to comprise a "movement impairment subscale." Each "yes" answer contributes one point to the score.

## Genotyping methods

### DNA samples

Genomic DNA was extracted from lymphoblastoid cell lines using a salting out protocol [35]. Additional DNA samples were obtained from saliva samples collected using Oragene self-collection kits and the DNA extracted according to the manufacturer's recommendation.

### Selection of genetic markers

One hundred twenty-one SNPs in the 14 genes studied were selected based on the haplotypes reconstructed by Haploview [36] or SNPHAP [37] with HapMap project genotype data. A SNP selection pipeline based on a double classification tree search algorithm [38] was used to capture the haplotype complexities and the tag SNPs (seleceted with Haploview for haplotype blocks).

### Genotyping

We genotyped the DNAs using Illumina GoldenGate chemistry on Sentrix Universal-96 Arrays. The Illumina array used interrogates 1536 SNP simultaneously, using a custom primer assay design (GS-0007064-OPA). 500 ng of DNA per well was genotyped using standard Illumina protocols. Arrays were imaged using an Illumina BeadStation 500 GX and data analyzed using GenCall 6.2.0.4 and GTSreports v5.1.2.0 software (Illumina). Ten percent of DNA samples were run in duplicate in order to obtain an estimate of genotyping reproducibility. The overall error rate was <0.005. Average genotyping completion rate was >0.98. Hardy-Weinberg equilibrium for each marker was tested with Chi-square tests using the R package "genetics" [39] or exact tests [40]. No deviation from the expected Hardy-Weinberg equilibrium values were observed for any of the values analyzed.

### Inference of haplotypes

Haplotype phases – i.e., how the directly measured SNP alleles were distributed into two chromosomes in each patient – were inferred by the expectation – maximization (EM) algorithm (SAS/Genetics, Cary, North Carolina, USA).

### Statistical analysis

Multiple linear regression was applied to examine the association between movement and each SNP, adjusting for eight other variables: age, sex, SF-36 Vitality, SF-36 Emotional Role, nonsurgical vs. surgical treatment, workman's compensation, marital status, and neurological exam deficits. An additive genetic model was tested by recoding the three SNP genotypes as 0 for the homozygote of the common allele, 1 for the heterozygote, and 2 for homozygote of the uncommon allele. In the haplotype analysis, first haplotype phases were inferred using expectation-maximization (EM) algorithm, where the probability was assigned to each pair of haplotypes which each subject possessed. Then, stepwise regression [41] was applied to test the association between movement score and haplotypes; the linear regression model included all haplotypes with frequency greater than 1% and the eight covariates.

## List of abbreviations used

GABA: Gamma-aminobutyric acid

GTP: Guanosine triphosphate

NIDCR: National Institute of Dental and Craniofacial Research

## Competing interests

Drs. Max, Belfer, Wu, Kingman, Goldman, and Atlas are listed as coinventors on a patent application for the use of a diagnostic test for GCH1 polymorphisms to predict the level of chronic pain.

## Authors' contributions

BKM carried out the review of motor neurochemistry, prioritized the polymorphisms, and cowrote the manuscript. MBM conceived of the project and analysis methods and cowrote the manuscript. TW did the statistical analyses, with advice of AK. CAH, IB, and CK carried out the genotyping. QY, CAH, and DG designed the genotyping array. DG provided senior scientific guidance on bioinformatics and genotyping issues. LGC provided advice on motor system physiology and clinical phenotype. SJA and RBK designed and carried out the original clinical study and advised on interpretation of the current analyses. All authors read and approved the final manuscript.

## Supplementary Material

Additional file 1 Association of SNPs in motor control candidate genes with movement scores in sciatica patients. Each SNP tested in the study is identified, with the number of subjects and mean movement limitation score for each genotype, and significance levels, assuming an additive model for allele effects. Click here for file

## References

[B1] Stewart WF, Ricci JA, Chee E, Morganstein D (2003). Lost productive work time costs from health conditions in the United States: results from the American Productivity Audit. J Occup Environ Med.

[B2] Deyo RA, Diehl AK, Rosenthal M (1986). How many days of bed rest for acute low back pain? A randomized clinical trial. N Engl J Med.

[B3] Malmivaara A, Hakkinen U, Aro T, Heinrichs ML, Koskenniemi L, Kuosma E, Lappi S, Paloheimo R, Servo C, Vaaranen V (1995). The treatment of acute low back pain – bed rest, exercises, or ordinary activity?. N Engl J Med.

[B4] Waddell G, Feder G, Lewis M (1997). Systematic reviews of bed rest and advice to stay active for acute low back pain. Br J Gen Pract.

[B5] Hagen KB, Jamtvedt G, Hilde G, Winnem MF (2005). The updated cochrane review of bed rest for low back pain and sciatica. Spine.

[B6] (CSAG) CSAG (1994). Report of a CSAG Committee on Back Pain.

[B7] Bigos SJ, United States. Agency for Health Care Policy and Research (1994). Acute low back problems in adults.

[B8] Lethem J, Slade PD, Troup JD, Bentley G (1983). Outline of a Fear-Avoidance Model of exaggerated pain perception – I. Behav Res Ther.

[B9] Vlaeyen JW, Linton SJ (2000). Fear-avoidance and its consequences in chronic musculoskeletal pain: a state of the art. Pain.

[B10] Sieben JM, Portegijs PJ, Vlaeyen JW, Knottnerus JA (2005). Pain-related fear at the start of a new low back pain episode. Eur J Pain.

[B11] Lund JP, Donga R, Widmer CG, Stohler CS (1991). The pain-adaptation model: a discussion of the relationship between chronic musculoskeletal pain and motor activity. Can J Physiol Pharmacol.

[B12] Zubieta JK, Heitzeg MM, Smith YR, Bueller JA, Xu K, Xu Y, Koeppe RA, Stohler CS, Goldman D (2003). COMT val158met genotype affects mu-opioid neurotransmitter responses to a pain stressor. Science.

[B13] Diatchenko L, Nackley AG, Slade GD, Bhalang K, Belfer I, Max MB, Goldman D, Maixner W (2006). Catechol-O-methyltransferase gene polymorphisms are associated with multiple pain-evoking stimuli. Pain.

[B14] Tegeder I, Costigan M, Griffin RS, Abele A, Belfer I, Schmidt H, Ehnert C, Nejim J, Marian C, Scholz J (2006). GTP cyclohydrolase and tetrahydrobiopterin regulate pain sensitivity and persistence. Nat Med.

[B15] Max MB, Wu T, Atlas SJ, Edwards RR, Haythornthwaite JA, Bollettino AF, Hipp HS, McKnight CD, Osman IA, Crawford EN (2006). A clinical genetic method to identify mechanisms by which pain causes depression and anxiety. Mol Pain.

[B16] Deyo RA, Battie M, Beurskens AJ, Bombardier C, Croft P, Koes B, Malmivaara A, Roland M, Von Korff M, Waddell G (1998). Outcome measures for low back pain research. A proposal for standardized use. Spine.

[B17] Roland M, Morris R (1983). A study of the natural history of back pain. Part I: development of a reliable and sensitive measure of disability in low-back pain. Spine.

[B18] Fairbank JC, Couper J, Davies JB, O'Brien JP (1980). The Oswestry low back pain disability questionnaire. Physiotherapy.

[B19] Belfer I, Wu T, Kingman A, Krishnaraju RK, Goldman D, Max MB (2004). Candidate gene studies of human pain mechanisms: methods for optimizing choice of polymorphisms and sample size. Anesthesiology.

[B20] Rudolph U, Mohler H (2006). GABA-based therapeutic approaches: GABAA receptor subtype functions. Curr Opin Pharmacol.

[B21] Rowlett JK, Cook JM, Duke AN, Platt DM (2005). Selective antagonism of GABAA receptor subtypes: an in vivo approach to exploring the therapeutic and side effects of benzodiazepine-type drugs. CNS Spectr.

[B22] Collins AL, Ma D, Whitehead PL, Martin ER, Wright HH, Abramson RK, Hussman JP, Haines JL, Cuccaro ML, Gilbert JR (2006). Investigation of autism and GABA receptor subunit genes in multiple ethnic groups. Neurogenetics.

[B23] (2007). Genome-wide association study of 14,000 cases of seven common diseases and 3,000 shared controls. Nature.

[B24] Neumeister A, Charney DS, Belfer I, Geraci M, Holmes C, Sharabi Y, Alim T, Bonne O, Luckenbaugh DA, Manji H (2005). Sympathoneural and adrenomedullary functional effects of alpha2C-adrenoreceptor gene polymorphism in healthy humans. Pharmacogenet Genomics.

[B25] Neumeister A, Drevets WC, Belfer I, Luckenbaugh DA, Henry S, Bonne O, Herscovitch P, Goldman D, Charney DS (2006). Effects of a alpha 2C-adrenoreceptor gene polymorphism on neural responses to facial expressions in depression. Neuropsychopharmacology.

[B26] Wagstaff AJ, Bryson HM (1997). Tizanidine. A review of its pharmacology, clinical efficacy and tolerability in the management of spasticity associated with cerebral and spinal disorders. Drugs.

[B27] Kleim JA, Chan S, Pringle E, Schallert K, Procaccio V, Jimenez R, Cramer SC (2006). BDNF val66met polymorphism is associated with modified experience-dependent plasticity in human motor cortex. Nat Neurosci.

[B28] Max MB, Gilron I, Loeser JD (2001). Antidepressants, muscle relaxants, and N-methyl-D-aspartate receptor antagonists. Bonica's Management of Pain.

[B29] Ciubotariu A, Arendt-Nielsen L, Graven-Nielsen T (2007). Localized muscle pain causes prolonged recovery after fatiguing isometric contractions. Exp Brain Res.

[B30] Moseley GL, Hodges PW (2005). Are the changes in postural control associated with low back pain caused by pain interference?. Clin J Pain.

[B31] Kong WZ, Goel VK, Gilbertson LG, Weinstein JN (1996). Effects of muscle dysfunction on lumbar spine mechanics. A finite element study based on a two motion segments model. Spine.

[B32] Panjabi MM (1992). The stabilizing system of the spine. Part I. Function, dysfunction, adaptation, and enhancement. J Spinal Disord.

[B33] Atlas SJ, Keller RB, Chang Y, Deyo RA, Singer DE (2001). Surgical and nonsurgical management of sciatica secondary to a lumbar disc herniation: five-year outcomes from the Maine Lumbar Spine Study. Spine.

[B34] Ware JE, Sherbourne CD (1992). The MOS 36-item short-form health survey (SF-36). I. Conceptual framework and item selection. Med Care.

[B35] Miller SA, Dykes DD, Polesky HF (1988). A simple salting out procedure for extracting DNA from human nucleated cells. Nucleic Acids Res.

[B36] Barrett JC, Fry B, Maller J, Daly MJ (2005). Haploview: analysis and visualization of LD and haplotype maps. Bioinformatics.

[B37] SNPHAP. http://www-gene.cimr.cam.ac.uk/clayton/software/snphap.txt.

[B38] Zhang P, Sheng H, Uehara R (2004). A double classification tree search algorithm for index SNP selection. BMC Bioinformatics.

[B39] R package, "genetics". http://cran.r-project.org/src/contrib/Descriptions/genetics.html.

[B40] Wigginton JE, Cutler DJ, Abecasis GR (2005). A note on exact tests of Hardy-Weinberg equilibrium. Am J Hum Genet.

[B41] Zaykin DV, Westfall PH, Young SS, Karnoub MA, Wagner MJ, Ehm MG (2002). Testing association of statistically inferred haplotypes with discrete and continuous traits in samples of unrelated individuals. Hum Hered.

[B42] Munafo MR, Johnstone EC, Welsh KI, Walton RT (2005). Association between the DRD2 gene Taq1A (C32806T) polymorphism and alcohol consumption in social drinkers. Pharmacogenomics J.

[B43] Elmslie FV, Hutchings SM, Spencer V, Curtis A, Covanis T, Gardiner RM, Rees M (1996). Analysis of GLRA1 in hereditary and sporadic hyperekplexia: a novel mutation in a family cosegregating for hyperekplexia and spastic paraparesis. J Med Genet.

[B44] Carsana A, Fortunato G, De Sarno C, Brancadoro V, Salvatore F (2003). Identification of new polymorphisms in the CACNA1S gene. Clin Chem Lab Med.

[B45] Horiuchi Y, Nakayama J, Ishiguro H, Ohtsuki T, Detera-Wadleigh SD, Toyota T, Yamada K, Nankai M, Shibuya H, Yoshikawa T (2004). Possible association between a haplotype of the GABA-A receptor alpha 1 subunit gene (GABRA1) and mood disorders. Biol Psychiatry.

[B46] Gillard EF, Otsu K, Fujii J, Duff C, de Leon S, Khanna VK, Britt BA, Worton RG, MacLennan DH (1992). Polymorphisms and deduced amino acid substitutions in the coding sequence of the ryanodine receptor (RYR1) gene in individuals with malignant hyperthermia. Genomics.

[B47] Feng J, Zheng J, Gelernter J, Kranzler H, Cook E, Goldman D, Jones IR, Craddock N, Heston LL, Delisi L (2001). An in-frame deletion in the alpha(2C) adrenergic receptor is common in African-Americans. Mol Psychiatry.

[B48] Polesskaya OO, Aston C, Sokolov BP (2006). Allele C-specific methylation of the 5-HT2A receptor gene: evidence for correlation with its expression and expression of DNA methylase DNMT1. J Neurosci Res.

[B49] Hisama FM, Gruen JR, Choi J, Huseinovic M, Grigorenko EL, Pauls D, Mattson RH, Gelernter J, Wood FB, Goei VL (2001). Human GABA(B) receptor 1 gene: eight novel sequence variants. Hum Mutat.

[B50] Roman T, Schmitz M, Polanczyk GV, Eizirik M, Rohde LA, Hutz MH (2003). Is the alpha-2A adrenergic receptor gene (ADRA2A) associated with attention-deficit/hyperactivity disorder?. Am J Med Genet B Neuropsychiatr Genet.

[B51] Lemonde S, Du L, Bakish D, Hrdina P, Albert PR (2004). Association of the C(-1019)G 5-HT1A functional promoter polymorphism with antidepressant response. Int J Neuropsychopharmacol.

[B52] Schmidt LG, Samochowiec J, Finckh U, Fiszer-Piosik E, Horodnicki J, Wendel B, Rommelspacher H, Hoehe MR (2002). Association of a CB1 cannabinoid receptor gene (CNR1) polymorphism with severe alcohol dependence. Drug Alcohol Depend.

[B53] Lucas JL, DeYoung JA, Sadee W (2001). Single nucleotide polymorphisms of the human M1 muscarinic acetylcholine receptor gene. AAPS PharmSci.

[B54] Sons MS, Plomp JJ (2006). Rab3A deletion selectively reduces spontaneous neurotransmitter release at the mouse neuromuscular synapse. Brain Res.

[B55] Kim MK, Lee SH, Park MS, Kim BC, Cho KH, Lee MC, Kim JH, Kim SM (2004). Mutation screening in Korean hypokalemic periodic paralysis patients: a novel SCN4A Arg672Cys mutation. Neuromuscul Disord.

