# Supplementary material for: Do motor control genes contribute to interindividual variability in decreased movement in patients with pain?
Source: Mol Pain. 2007 Jul 26;3:20. doi: 10.1186/1744-8069-3-20 (PMC2072938; doi:10.1186/1744-8069-3-20)
Supplement: Additional file 1 — Association of SNPs in motor control candidate genes with movement scores in sciatica patients. Each SNP tested in the study is identified, with the number of subjects and mean movement limitation score for each genotype, and significance levels, assuming an additive model for allele effects. [file 1744-8069-3-20-S1.doc]

Supplementary Table 1. Association of SNPs in motor control candidate genes with movement scores in sciatica patients.

| **Gene** | **dbSNP ID** | **Allele_1** | **Allele_2** | **Rare allele** | **Genotype** | **COUNT** | **Mvmt Score** | ***P value*** |
| --- | --- | --- | --- | --- | --- | --- | --- | --- |
| 5HTR1A | rs1364043 | A | C | 2 | 1/1 | 188 | 11.11 | 0.6662 |
|  |  |  |  |  | 1/2 | 112 | 10.92 |  |
|  |  |  |  |  | 2/2 | 10 | 10.80 |  |
| 5HTR1A | rs1423691 | T | C | 1 | 1/1 | 69 | 10.72 | 0.5231 |
|  |  |  |  |  | 1/2 | 164 | 11.16 |  |
|  |  |  |  |  | 2/2 | 77 | 11.08 |  |
| 5HTR1A | rs878567 | T | C | 1 | 1/1 | 69 | 10.72 | 0.5231 |
|  |  |  |  |  | 1/2 | 164 | 11.16 |  |
|  |  |  |  |  | 2/2 | 77 | 11.08 |  |
| 5HTR1A | rs10042486 | T | C | 2 | 1/1 | 78 | 11.15 | 0.4299 |
|  |  |  |  |  | 1/2 | 163 | 11.12 |  |
|  |  |  |  |  | 2/2 | 69 | 10.71 |  |
| 5HTR2A | rs6314 | A | G | 1 | 1/1 | 5 | 11.14 | 0.4492 |
|  |  |  |  |  | 1/2 | 53 | 11.30 |  |
|  |  |  |  |  | 2/2 | 252 | 10.96 |  |
| 5HTR2A | rs1923882 | A | G | 1 | 1/1 | 17 | 10.77 | 0.8475 |
|  |  |  |  |  | 1/2 | 119 | 11.12 |  |
|  |  |  |  |  | 2/2 | 174 | 10.99 |  |
| 5HTR2A | rs9567735 | T | C | 2 | 1/1 | 241 | 11.07 | 0.6661 |
|  |  |  |  |  | 1/2 | 68 | 10.87 |  |
| 5HTR2A | rs6561333 | A | G | 2 | 1/1 | 82 | 11.47 | 0.3731 |
|  |  |  |  |  | 1/2 | 152 | 10.83 |  |
|  |  |  |  |  | 2/2 | 76 | 11.03 |  |
| 5HTR2A | rs2296972 | A | C | 1 | 1/1 | 40 | 12.11 | 0.1324 |
|  |  |  |  |  | 1/2 | 131 | 10.88 |  |
|  |  |  |  |  | 2/2 | 139 | 10.95 |  |
| 5HTR2A | rs9316233 | C | G | 1 | 1/1 | 13 | 11.81 | 0.1368 |
|  |  |  |  |  | 1/2 | 88 | 11.30 |  |
|  |  |  |  |  | 2/2 | 209 | 10.84 |  |
| 5HTR2A | rs1928042 | A | C | 2 | 1/1 | 155 | 11.03 | 0.2894 |
|  |  |  |  |  | 1/2 | 133 | 10.90 |  |
|  |  |  |  |  | 2/2 | 22 | 12.48 |  |
| 5HTR2A | rs582385 | A | G | 2 | 1/1 | 189 | 11.07 | 0.7420 |
|  |  |  |  |  | 1/2 | 106 | 10.86 |  |
|  |  |  |  |  | 2/2 | 15 | 11.92 |  |
| 5HTR2A | rs1928040 | A | G | 1 | 1/1 | 43 | 10.91 | 0.6652 |
|  |  |  |  |  | 1/2 | 173 | 11.03 |  |
|  |  |  |  |  | 2/2 | 94 | 11.09 |  |
| 5HTR2A | rs731779 | T | G | 2 | 1/1 | 194 | 10.93 | 0.7691 |
|  |  |  |  |  | 1/2 | 104 | 11.21 |  |
|  |  |  |  |  | 2/2 | 12 | 9.60 |  |
| 5HTR2A | rs985933 | T | C | 1 | 1/1 | 47 | 11.55 | 0.6437 |
|  |  |  |  |  | 1/2 | 149 | 10.81 |  |
|  |  |  |  |  | 2/2 | 114 | 11.13 |  |
| 5HTR2A | rs927544 | A | G | 2 | 1/1 | 152 | 11.02 | 0.9653 |
|  |  |  |  |  | 1/2 | 134 | 11.08 |  |
|  |  |  |  |  | 2/2 | 24 | 10.81 |  |
| 5HTR2A | rs9534505 | T | C | 1 | 1/1 | 2 | 13.59 | 0.7133 |
|  |  |  |  |  | 1/2 | 51 | 10.95 |  |
|  |  |  |  |  | 2/2 | 257 | 11.04 |  |
| 5HTR2A | rs4941573 | A | G | 2 | 1/1 | 114 | 11.19 | 0.3312 |
|  |  |  |  |  | 1/2 | 159 | 10.98 |  |
|  |  |  |  |  | 2/2 | 37 | 10.70 |  |
| 5HTR2A | rs1328685 | T | C | 2 | 1/1 | 251 | 10.98 | 0.4517 |
|  |  |  |  |  | 1/2 | 54 | 11.05 |  |
|  |  |  |  |  | 2/2 | 5 | 12.66 |  |
| 5HTR2A | rs4142900 | T | G | 2 | 1/1 | 73 | 11.35 | 0.1617 |
|  |  |  |  |  | 1/2 | 171 | 11.07 |  |
|  |  |  |  |  | 2/2 | 66 | 10.65 |  |
| ADRA1C | rs2291776 | A | G | 1 | 1/2 | 46 | 10.74 | 0.6546 |
|  |  |  |  |  | 2/2 | 263 | 11.09 |  |
| ADRA1C | rs2036109 | A | C | 1 | 1/1 | 50 | 10.70 | 0.9420 |
|  |  |  |  |  | 1/2 | 151 | 11.21 |  |
|  |  |  |  |  | 2/2 | 109 | 10.92 |  |
| ADRA1C | rs10102186 | A | G | 1 | 1/1 | 38 | 10.64 | 0.6433 |
|  |  |  |  |  | 1/2 | 138 | 11.10 |  |
|  |  |  |  |  | 2/2 | 134 | 11.07 |  |
| ADRA1C | rs1048101 | A | G | 2 | 1/1 | 81 | 11.04 | 0.9211 |
|  |  |  |  |  | 1/2 | 152 | 11.08 |  |
|  |  |  |  |  | 2/2 | 77 | 10.92 |  |
| ADRA1C | rs11135955 | A | C | 2 | 1/1 | 239 | 11.01 | 0.6952 |
|  |  |  |  |  | 1/2 | 65 | 11.15 |  |
|  |  |  |  |  | 2/2 | 6 | 9.48 |  |
| ADRA1C | rs2055195 | A | G | 2 | 1/1 | 82 | 11.02 | 0.9620 |
|  |  |  |  |  | 1/2 | 148 | 11.03 |  |
|  |  |  |  |  | 2/2 | 80 | 11.05 |  |
| ADRA1C | rs7016881 | T | A | 1 | 1/2 | 42 | 10.71 | 0.5916 |
|  |  |  |  |  | 2/2 | 268 | 11.09 |  |
| ADRA1C | rs2036108 | A | G | 1 | 1/1 | 23 | 11.23 | 0.8839 |
|  |  |  |  |  | 1/2 | 115 | 10.94 |  |
|  |  |  |  |  | 2/2 | 172 | 11.06 |  |
| ADRA1C | rs11781115 | T | G | 2 | 1/1 | 134 | 11.23 | 0.2388 |
|  |  |  |  |  | 1/2 | 144 | 10.99 |  |
|  |  |  |  |  | 2/2 | 32 | 10.58 |  |
| ADRA1C | rs526302 | T | G | 1 | 1/1 | 20 | 12.72 | 0.0752 |
|  |  |  |  |  | 1/2 | 106 | 11.02 |  |
|  |  |  |  |  | 2/2 | 184 | 10.84 |  |
| ADRA1C | rs472151 | A | G | 1 | 1/1 | 71 | 11.42 | 0.7146 |
|  |  |  |  |  | 1/2 | 157 | 10.68 |  |
|  |  |  |  |  | 2/2 | 82 | 11.24 |  |
| ADRA1C | rs10503800 | T | G | 1 | 1/1 | 32 | 11.46 | 0.4150 |
|  |  |  |  |  | 1/2 | 144 | 11.03 |  |
|  |  |  |  |  | 2/2 | 134 | 10.92 |  |
| ADRA1C | rs2046186 | T | C | 2 | 1/1 | 198 | 11.05 | 0.9127 |
|  |  |  |  |  | 1/2 | 104 | 10.88 |  |
|  |  |  |  |  | 2/2 | 8 | 12.13 |  |
| ADRA1C | rs2644627 | C | G | 1 | 1/1 | 61 | 11.48 | 0.7819 |
|  |  |  |  |  | 1/2 | 153 | 10.79 |  |
|  |  |  |  |  | 2/2 | 96 | 11.16 |  |
| ADRA2A | rs638019 | T | C | 1 | 1/1 | 25 | 12.13 | 0.4450 |
|  |  |  |  |  | 1/2 | 108 | 10.73 |  |
|  |  |  |  |  | 2/2 | 177 | 11.03 |  |
| ADRA2A | rs1800544 | G | C | 1 | 1/1 | 24 | 12.35 | 0.4306 |
|  |  |  |  |  | 1/2 | 107 | 10.65 |  |
|  |  |  |  |  | 2/2 | 179 | 11.05 |  |
| ADRA2C | rs13118771 | T | C | 2 | 1/1 | 253 | 11.22 | 0.0267 |
|  |  |  |  |  | 1/2 | 53 | 10.16 |  |
|  |  |  |  |  | 2/2 | 4 | 9.87 |  |
| ADRA2C | rs6846820 | A | G | 1 | 1/1 | 6 | 9.19 | 0.0182 |
|  |  |  |  |  | 1/2 | 51 | 10.26 |  |
|  |  |  |  |  | 2/2 | 253 | 11.23 |  |
| ADRA2C | rs7434444 | C | G | 1 | 1/1 | 16 | 11.63 | 0.4313 |
|  |  |  |  |  | 1/2 | 139 | 11.13 |  |
|  |  |  |  |  | 2/2 | 155 | 10.88 |  |
| ADRA2C | rs7678463 | C | G | 1 | 1/1 | 6 | 9.35 | 0.2490 |
|  |  |  |  |  | 1/2 | 74 | 10.84 |  |
|  |  |  |  |  | 2/2 | 230 | 11.12 |  |
| CHRM1 | rs17157628 | A | G | 1 | 1/1 | 2 | 8.88 | 0.8893 |
|  |  |  |  |  | 1/2 | 44 | 11.37 |  |
|  |  |  |  |  | 2/2 | 264 | 11.01 |  |
| CHRM1 | rs544978 | A | C | 2 | 1/1 | 140 | 11.33 | 0.3526 |
|  |  |  |  |  | 1/2 | 140 | 10.64 |  |
|  |  |  |  |  | 2/2 | 30 | 11.41 |  |
| CHRM1 | rs542269 | A | G | 2 | 1/1 | 139 | 11.33 | 0.3502 |
|  |  |  |  |  | 1/2 | 141 | 10.64 |  |
|  |  |  |  |  | 2/2 | 30 | 11.40 |  |
| CHRM1 | rs2075748 | T | C | 1 | 1/1 | 18 | 12.04 | 0.2234 |
|  |  |  |  |  | 1/2 | 120 | 11.04 |  |
|  |  |  |  |  | 2/2 | 172 | 10.93 |  |
| CNR1 | rs1049353 | A | G | 1 | 1/1 | 29 | 11.09 | 0.7509 |
|  |  |  |  |  | 1/2 | 117 | 11.12 |  |
|  |  |  |  |  | 2/2 | 164 | 10.96 |  |
| CNR1 | rs806377 | T | C | 1 | 1/1 | 74 | 11.30 | 0.3082 |
|  |  |  |  |  | 1/2 | 154 | 11.00 |  |
|  |  |  |  |  | 2/2 | 82 | 10.81 |  |
| CNR1 | rs806378 | T | C | 1 | 1/1 | 30 | 11.40 | 0.7961 |
|  |  |  |  |  | 1/2 | 109 | 10.80 |  |
|  |  |  |  |  | 2/2 | 171 | 11.13 |  |
| CNR1 | rs806381 | T | C | 2 | 1/1 | 150 | 11.17 | 0.6331 |
|  |  |  |  |  | 1/2 | 122 | 10.80 |  |
|  |  |  |  |  | 2/2 | 38 | 11.23 |  |
| CNR1 | rs6454674 | T | G | 2 | 1/1 | 152 | 11.13 | 0.6050 |
|  |  |  |  |  | 1/2 | 128 | 10.90 |  |
|  |  |  |  |  | 2/2 | 30 | 11.07 |  |
| CNR1 | rs9344757 | T | G | 1 | 1/1 | 34 | 11.23 | 0.8280 |
|  |  |  |  |  | 1/2 | 130 | 10.86 |  |
|  |  |  |  |  | 2/2 | 146 | 11.17 |  |
| DRD2 | rs2242592 | T | C | 2 | 1/1 | 155 | 11.02 | 0.9565 |
|  |  |  |  |  | 1/2 | 126 | 11.06 |  |
|  |  |  |  |  | 2/2 | 29 | 10.92 |  |
| DRD2 | rs2587548 | C | G | 2 | 1/1 | 123 | 11.28 | 0.3833 |
|  |  |  |  |  | 1/2 | 142 | 10.87 |  |
|  |  |  |  |  | 2/2 | 45 | 10.95 |  |
| DRD2 | rs1076563 | T | G | 1 | 1/1 | 45 | 10.95 | 0.3698 |
|  |  |  |  |  | 1/2 | 141 | 10.85 |  |
|  |  |  |  |  | 2/2 | 124 | 11.29 |  |
| DRD2 | rs1079596 | T | C | 1 | 1/1 | 6 | 10.04 | 0.2562 |
|  |  |  |  |  | 1/2 | 77 | 10.79 |  |
|  |  |  |  |  | 2/2 | 227 | 11.17 |  |
| DRD2 | rs1125394 | A | G | 2 | 1/1 | 227 | 11.17 | 0.2562 |
|  |  |  |  |  | 1/2 | 77 | 10.79 |  |
|  |  |  |  |  | 2/2 | 6 | 10.04 |  |
| DRD2 | rs2471857 | A | G | 1 | 1/1 | 6 | 10.04 | 0.2562 |
|  |  |  |  |  | 1/2 | 77 | 10.79 |  |
|  |  |  |  |  | 2/2 | 227 | 11.17 |  |
| DRD2 | rs7103679 | T | C | 1 | 1/1 | 4 | 9.80 | 0.2875 |
|  |  |  |  |  | 1/2 | 80 | 10.83 |  |
|  |  |  |  |  | 2/2 | 226 | 11.17 |  |
| DRD2 | rs4648318 | T | C | 2 | 1/1 | 185 | 11.10 | 0.7251 |
|  |  |  |  |  | 1/2 | 108 | 10.91 |  |
|  |  |  |  |  | 2/2 | 17 | 11.13 |  |
| DRD2 | rs4274224 | A | G | 2 | 1/1 | 93 | 11.48 | 0.2857 |
|  |  |  |  |  | 1/2 | 155 | 10.85 |  |
|  |  |  |  |  | 2/2 | 62 | 10.90 |  |
| DRD2 | rs4581480 | T | C | 2 | 1/1 | 259 | 11.07 | 0.7248 |
|  |  |  |  |  | 1/2 | 51 | 10.86 |  |
| DRD2 | rs4648317 | T | C | 1 | 1/1 | 11 | 11.34 | 0.8667 |
|  |  |  |  |  | 1/2 | 90 | 11.07 |  |
|  |  |  |  |  | 2/2 | 209 | 11.01 |  |
| DRD2 | rs4350392 | T | G | 1 | 1/1 | 11 | 11.35 | 0.6380 |
|  |  |  |  |  | 1/2 | 92 | 11.18 |  |
|  |  |  |  |  | 2/2 | 207 | 10.97 |  |
| DRD2 | rs12364283 | A | G | 2 | 1/1 | 249 | 10.96 | 0.2747 |
|  |  |  |  |  | 1/2 | 56 | 11.58 |  |
|  |  |  |  |  | 2/2 | 5 | 11.08 |  |
| GABRA2 | rs693547 | T | A | 1 | 1/1 | 58 | 10.85 | 0.5881 |
|  |  |  |  |  | 1/2 | 156 | 11.07 |  |
|  |  |  |  |  | 2/2 | 96 | 11.11 |  |
| GABRA2 | rs519270 | A | G | 1 | 1/1 | 57 | 10.76 | 0.4610 |
|  |  |  |  |  | 1/2 | 156 | 11.10 |  |
|  |  |  |  |  | 2/2 | 97 | 11.14 |  |
| GABRA2 | rs279847 | A | C | 1 | 1/1 | 57 | 10.74 | 0.4922 |
|  |  |  |  |  | 1/2 | 159 | 11.12 |  |
|  |  |  |  |  | 2/2 | 94 | 11.13 |  |
| GABRA2 | rs279843 | A | G | 1 | 1/1 | 59 | 10.69 | 0.3816 |
|  |  |  |  |  | 1/2 | 156 | 11.13 |  |
|  |  |  |  |  | 2/2 | 95 | 11.17 |  |
| GABRA2 | rs279827 | A | G | 2 | 1/1 | 93 | 11.29 | 0.1659 |
|  |  |  |  |  | 1/2 | 159 | 11.12 |  |
|  |  |  |  |  | 2/2 | 58 | 10.55 |  |
| GABRA2 | rs426463 | T | G | 2 | 1/1 | 93 | 11.30 | 0.1857 |
|  |  |  |  |  | 1/2 | 158 | 11.11 |  |
|  |  |  |  |  | 2/2 | 59 | 10.59 |  |
| GABRA2 | rs10805145 | T | C | 2 | 1/1 | 93 | 11.30 | 0.1857 |
|  |  |  |  |  | 1/2 | 158 | 11.11 |  |
|  |  |  |  |  | 2/2 | 59 | 10.59 |  |
| GABRA2 | rs1442060 | A | G | 1 | 1/1 | 68 | 11.26 | 0.6025 |
|  |  |  |  |  | 1/2 | 150 | 10.95 |  |
|  |  |  |  |  | 2/2 | 92 | 10.97 |  |
| GABRA2 | rs9291283 | T | C | 1 | 1/1 | 17 | 11.63 | 0.9369 |
|  |  |  |  |  | 1/2 | 117 | 10.91 |  |
|  |  |  |  |  | 2/2 | 176 | 11.10 |  |
| GABRA2 | rs11503014 | C | G | 1 | 1/1 | 16 | 11.43 | 0.8362 |
|  |  |  |  |  | 1/2 | 120 | 10.86 |  |
|  |  |  |  |  | 2/2 | 174 | 11.12 |  |
| GABRB1 | rs2236781 | G | C | 1 | 1/1 | 79 | 11.28 | 0.3964 |
|  |  |  |  |  | 1/2 | 143 | 11.00 |  |
|  |  |  |  |  | 2/2 | 88 | 10.80 |  |
| GABRB1 | rs4315750 | A | C | 1 | 1/1 | 4 | 13.01 | 0.4716 |
|  |  |  |  |  | 1/2 | 38 | 11.04 |  |
|  |  |  |  |  | 2/2 | 268 | 11.00 |  |
| GABRB1 | rs971353 | A | G | 2 | 1/1 | 178 | 11.10 | 0.9430 |
|  |  |  |  |  | 1/2 | 120 | 10.85 |  |
|  |  |  |  |  | 2/2 | 12 | 12.00 |  |
| GABRB1 | rs6447532 | G | C | 1 | 1/1 | 38 | 11.28 | 0.9164 |
|  |  |  |  |  | 1/2 | 132 | 10.86 |  |
|  |  |  |  |  | 2/2 | 140 | 11.12 |  |
| GABRB1 | rs7666487 | C | G | 2 | 1/1 | 142 | 11.25 | 0.8850 |
|  |  |  |  |  | 1/2 | 132 | 10.72 |  |
|  |  |  |  |  | 2/2 | 36 | 11.53 |  |
| GABRB1 | rs3114088 | T | C | 1 | 1/1 | 37 | 11.52 | 0.9784 |
|  |  |  |  |  | 1/2 | 133 | 10.77 |  |
|  |  |  |  |  | 2/2 | 140 | 11.20 |  |
| GABRB1 | rs10026884 | A | G | 2 | 1/1 | 140 | 11.09 | 0.3286 |
|  |  |  |  |  | 1/2 | 128 | 11.25 |  |
|  |  |  |  |  | 2/2 | 42 | 10.22 |  |
| GABRB1 | rs9996854 | T | C | 2 | 1/1 | 112 | 10.98 | 0.8475 |
|  |  |  |  |  | 1/2 | 152 | 11.10 |  |
|  |  |  |  |  | 2/2 | 46 | 10.78 |  |
| GABRB1 | rs4694846 | A | G | 2 | 1/1 | 125 | 11.35 | 0.1862 |
|  |  |  |  |  | 1/2 | 136 | 10.85 |  |
|  |  |  |  |  | 2/2 | 49 | 10.81 |  |
| GABRB1 | rs17461905 | A | G | 2 | 1/1 | 222 | 10.83 | 0.1219 |
|  |  |  |  |  | 1/2 | 84 | 11.46 |  |
|  |  |  |  |  | 2/2 | 4 | 11.73 |  |
| GABRB1 | rs13107066 | A | C | 2 | 1/1 | 103 | 11.17 | 0.8874 |
|  |  |  |  |  | 1/2 | 136 | 10.84 |  |
|  |  |  |  |  | 2/2 | 71 | 11.09 |  |
| GABRB1 | rs6813436 | A | G | 1 | 1/1 | 4 | 10.53 | 0.6383 |
|  |  |  |  |  | 1/2 | 68 | 11.29 |  |
|  |  |  |  |  | 2/2 | 238 | 10.96 |  |
| GABRB1 | rs7439087 | A | G | 2 | 1/1 | 148 | 10.73 | 0.2294 |
|  |  |  |  |  | 1/2 | 131 | 11.29 |  |
|  |  |  |  |  | 2/2 | 31 | 11.19 |  |
| GABRB1 | rs6290 | T | C | 1 | 1/2 | 42 | 10.93 | 0.8447 |
|  |  |  |  |  | 2/2 | 268 | 11.04 |  |
| GABRB1 | rs7679148 | A | C | 1 | 1/1 | 23 | 10.78 | 0.2766 |
|  |  |  |  |  | 1/2 | 120 | 10.81 |  |
|  |  |  |  |  | 2/2 | 167 | 11.22 |  |
| GABRB1 | rs4591574 | A | G | 2 | 1/1 | 100 | 10.89 | 0.9083 |
|  |  |  |  |  | 1/2 | 147 | 11.32 |  |
|  |  |  |  |  | 2/2 | 63 | 10.74 |  |
| GABRB1 | rs10028945 | A | G | 1 | 1/1 | 22 | 10.91 | 0.4367 |
|  |  |  |  |  | 1/2 | 119 | 10.85 |  |
|  |  |  |  |  | 2/2 | 169 | 11.17 |  |
| GLRA1 | rs2915885 | T | C | 2 | 1/1 | 107 | 10.93 | 0.2468 |
|  |  |  |  |  | 1/2 | 144 | 10.88 |  |
|  |  |  |  |  | 2/2 | 59 | 11.60 |  |
| GLRA1 | rs11167557 | A | G | 2 | 1/1 | 107 | 10.93 | 0.2468 |
|  |  |  |  |  | 1/2 | 144 | 10.88 |  |
|  |  |  |  |  | 2/2 | 59 | 11.60 |  |
| GLRA1 | rs4075273 | T | G | 1 | 1/1 | 59 | 11.60 | 0.2716 |
|  |  |  |  |  | 1/2 | 143 | 10.87 |  |
|  |  |  |  |  | 2/2 | 108 | 10.95 |  |
| GLRA1 | rs9324714 | A | T | 1 | 1/1 | 59 | 11.60 | 0.2675 |
|  |  |  |  |  | 1/2 | 142 | 10.87 |  |
|  |  |  |  |  | 2/2 | 109 | 10.95 |  |
| GLRA1 | rs1428159 | T | C | 2 | 1/1 | 108 | 10.97 | 0.2921 |
|  |  |  |  |  | 1/2 | 143 | 10.85 |  |
|  |  |  |  |  | 2/2 | 59 | 11.60 |  |
| GLRA1 | rs1346489 | A | G | 1 | 1/1 | 16 | 12.56 | 0.8783 |
|  |  |  |  |  | 1/2 | 121 | 10.69 |  |
|  |  |  |  |  | 2/2 | 173 | 11.19 |  |
| GLRA1 | rs2964608 | A | G | 1 | 1/1 | 40 | 10.74 | 0.1096 |
|  |  |  |  |  | 1/2 | 151 | 10.69 |  |
|  |  |  |  |  | 2/2 | 119 | 11.48 |  |
| GLRA1 | rs1428155 | A | G | 2 | 1/1 | 118 | 11.41 | 0.1710 |
|  |  |  |  |  | 1/2 | 150 | 10.71 |  |
|  |  |  |  |  | 2/2 | 42 | 10.73 |  |
| GLRA1 | rs991738 | A | G | 1 | 1/1 | 59 | 11.46 | 0.5879 |
|  |  |  |  |  | 1/2 | 160 | 10.83 |  |
|  |  |  |  |  | 2/2 | 91 | 11.00 |  |
| GLRA1 | rs1428157 | A | C | 1 | 1/1 | 35 | 10.61 | 0.8929 |
|  |  |  |  |  | 1/2 | 125 | 11.28 |  |
|  |  |  |  |  | 2/2 | 150 | 10.88 |  |
| TAC1 | rs6465606 | T | C | 1 | 1/1 | 24 | 9.80 | 0.0713 |
|  |  |  |  |  | 1/2 | 112 | 10.87 |  |
|  |  |  |  |  | 2/2 | 174 | 11.24 |  |
| TAC1 | rs2072100 | T | C | 2 | 1/1 | 88 | 10.79 | 0.9603 |
|  |  |  |  |  | 1/2 | 148 | 11.23 |  |
|  |  |  |  |  | 2/2 | 74 | 10.76 |  |
| TAC1 | rs1229434 | A | G | 2 | 1/1 | 108 | 10.97 | 0.7707 |
|  |  |  |  |  | 1/2 | 143 | 11.02 |  |
|  |  |  |  |  | 2/2 | 59 | 11.16 |  |
| TAC1 | rs12532490 | A | G | 2 | 1/1 | 206 | 10.82 | 0.0687 |
|  |  |  |  |  | 1/2 | 93 | 11.53 |  |
|  |  |  |  |  | 2/2 | 11 | 11.74 |  |
| TH | rs6356 | A | G | 1 | 1/1 | 50 | 10.88 | 0.9037 |
|  |  |  |  |  | 1/2 | 134 | 11.16 |  |
|  |  |  |  |  | 2/2 | 126 | 10.94 |  |
| TH | rs2070762 | A | G | 1 | 1/1 | 69 | 11.11 | 0.3679 |
|  |  |  |  |  | 1/2 | 163 | 10.71 |  |
|  |  |  |  |  | 2/2 | 78 | 11.57 |  |

*p* values shown are for additive model, which assumes that each copy of the uncommon allele alters the movement score by a similar amount.
